# Polycaprolactone for the Correction of Nasolabial Folds: A 24-Month, Prospective, Randomized, Controlled Clinical Trial

**DOI:** 10.1111/dsu.12054

**Published:** 2013-01-25

**Authors:** Marion Michaela Moers-Carpi, Sally Sherwood

**Affiliations:** * Private Clinic, HautokMunich, Germany; † Sally Sherwood CommunicationsNew York, New York

## Abstract

**Background:**

In this study, we examined two polycaprolactone (PCL)-based dermal filler formulas (PCL-1; PCL-2) for safety, patient satisfaction, likelihood to return, efficacy, and duration of correction.

**Objective:**

This 40-patient, 24-month, prospective, randomized, controlled study evaluated the efficacy, safety, longevity, and volume of two PCL formulas for correction of nasolabial folds.

**Methods:**

Patients enrolled in a medical clinic in Europe received two injections 1 month apart and returned at 3, 6, 9, 12, 15, 18, and 24 months for blinded patient evaluation using accepted aesthetic rating scales.

**Results:**

At 12 months, the efficacy outcomes on Wrinkle Severity Rating Scale (WSRS) and Global Aesthetic Improvement Scale (GAIS) of PCL-1 and PCL-2 were consistently maintained, with sustained improvement in 90% and 91.4% of patients, respectively. At 24 months, PCL-2 was found to be more effective than PCL-1 with respect to GAIS and WSRS, showing sustained improvement for the entire 2-year study period (linear *p* = .52; quadratic *p* > .99). Patient satisfaction at 24 months was 72.4% for PCL-1 and 81.7% for PCL-2. Both products were found to be safe and well tolerated.

**Conclusions:**

PCL-1 and PCL-2 are safe and have sustained efficacy and high patient satisfaction, with PCL-2 demonstrating longer-lasting results than PCL-1.

Injectable fillers have become an increasingly popular option in the treatment for aesthetic facial enhancements and appeal to the growing population wanting to reverse the signs of aging. With recent technologic advances, newer types of dermal fillers have been approved, providing practitioners the option of administering soft tissue fillers–such as hyaluronic acid and calcium hydroxylapatite (CaHA)–with minimal inconvenience to the patient, although some shortcomings remain unaddressed.

Over the last decades, the safety and efficacy of dermal fillers have improved continuously, with an ongoing quest for safe but longer-lasting although not permanent results.^1^ Moreover, in the growing market of noninvasive treatments for soft tissue augmentation, which are replacing surgical interventions, the search for improved therapies for correction of wrinkles and folds, contouring, sculpting, and volumizing is steadily improving.

## Polycaprolactone

This article details a clinical study in which two polycaprolactone (PCL)-based, biocompatible, long-lasting, bioresorbable soft tissue filler formulas (Ellansé-S [PCL-1] and Ellansé-M [PCL-2], AQTIS Medical BV, Utrecht, The Netherlands) were administered in a 24-month, prospective, randomized, controlled clinical trial.

These two formulas are part of a novel dermal filler family that incorporates sustained performance (continuous and stable improvement over time), tunable longevity (duration of clinical performance can be modified by adjusting the initial PCL formula), and total bioresorbability (complete and controlled bioresorption process) (STAT).

This dermal filler family is composed of smooth, soft, non-cross-linked bioresorbable PCL microspheres (25–50 μm) homogeneously suspended in an aqueous carboxymethylcellulose (CMC) gel carrier. All formulas are available in sterile ready-to-use, prefilled 1.0-mL syringes. PCL and CMC have been used successfully for decades in numerous Conformité Européene (CE)-marked and Food and Drug Adminstration–approved bioresorbable device applications in the medical, cosmetic, and pharmaceutical industries (e.g., oral and maxillofacial surgery, wound dressing, bioresorbable sutures, and controlled drug delivery).^2^–^7^ CMC is a well-known carrier for dermal fillers.^7^,^8^

The biocompatibility and in vivo behavior of PCL as a bioresorbable medical polymer have been well documented since the 1980s.^3^–^5^,^9^–^12^ Its bioresorption characteristics are attractive because of its controlled and safe bioresorption by the hydrolysis of the polymer ester-linkages, resulting in nontoxic bioresorption products that are resorbed through the normal metabolic pathways and readily excreted.^9^–^12^ The controlled bioresorption of PCL has been proven in^3^H- and C^14^-labeled PCL implantation studies.^9^,^10^

Dermal filler characteristics such as a particle size, particle size distribution, particle concentration, particle surface, shape, gel viscosity and elasticity, gel homogeneity, and injectability are the same throughout the family. Furthermore, the smooth, sphere shape of the microparticles and their size and concentration stimulate the formation of new high-quality collagen (neocollagenesis).^13^–^17^

The only distinguishing characteristic within the dermal filler family is the initial average length of the individual polymer chains within the microspheres, which is the basis for the different duration options within the dermal filler family as a result of their difference in bioresorption time. Of the two formulas used in this study, PCL-2 has a higher average initial polymer chain length than PCL-1 and as such a longer bioresorption time.

Upon injection, wrinkles and folds are immediately corrected because of the viscosity of the gel carrier and the presence of the microspheres. Macrophages gradually resorb the gel carrier over a period of several weeks, which patients' own new collagen replaces, creating a three-dimensional scaffold anchoring the microspheres.

In early 2009, the PCL-based dermal family received CE marking for deep dermal and subdermal implantation for the correction of wrinkles and folds. This is the first and currently only dermal filler that uses bioresorbable PCL microspheres for soft tissue augmentation.

## Methods

### Study Objective

The objective of this study was to evaluate the safety and effectiveness of two PCL formulas, PCL-1 and PCL-2, for the correction of nasolabial folds (NLFs).

### Patient Population

The study enrolled 40 subjects (38 women (95%) and two men (5%)) aged 36–69; 30 were treated with PCL-2 and 10 with PCL-1. Subjects were enrolled if they had a rating of 3 or 4 on the Wrinkle Severity Rating Scale (WSRS; moderate to severe NLFs).

### Design

In this two-arm study, subjects were randomized to receive the PCL-1 or PCL-2 formula for the correction of both NLFs. Both formulas were supplied in sterile ready-to-use, prefilled 1.0-mL syringes.

All subjects were intended to receive an initial treatment and were eligible for a touch-up treatment at 1 month to provide optimal correction. Because this was a first-in-human study, subjects were initially given a suboptimal dose, and the investigators were allowed to provide a touch-up at the 1-month follow-up visit. Injected volumes for initial and touch-up treatments were recorded for all subjects. Subjects then returned for physician and subject evaluation at 3, 6, 9, 12, 15, 18, and 24 months. The study was conducted in accordance with International Standards Organization 14155 and the International Conference on Harmonisation Good Clinical Practice E6, and the study protocol conformed to the guidelines of the 1975 Declaration of Helsinki. Ethics committee approval was obtained before study initiation.

Subjects had to be aged 18 and older with moderate to severe NLFs as determined according to a WSRS score of 3 or 4 in both folds at the pretreatment evaluation and be willing to abstain from other facial cosmetic procedures that could interfere with treatment outcomes through the 24-month follow-up visit (e.g., laser or chemical resurfacing, dermabrasion, botulinum toxin injections in or near the nasolabial area, aesthetic facial surgery, facial wrinkle treatments of the nasolabial area, lip enhancements), be willing and able to adhere to study follow-up procedures and schedule, and provide written informed consent for participation in the study

Subjects who had received previous permanent implants in the nasolabial area at any time or undergone any aesthetic facial procedure in the nasolabial area within 6 months before enrollment that could interfere with study results; who had been treated with chemotherapy agents or systemic corticosteroids within 3 months before enrollment; who had received antiplatelets, anticoagulants, thrombolytics, vitamin E, or anti-inflammatories 1 week before to 1 month after treatment; with a history of autoimmune disorder; with known allergies to topical or injectable anesthetics; with severe allergies manifested by a history of anaphylaxis or with severe, chronic allergies; with acute, chronic, or recurrent skin disease near the nasolabial area; with a known bleeding disorder; with an active infection of any kind at the time of enrollment; with known connective tissue disease; who were pregnant or lactating; and who were enrolled in another investigational clinical trial were excluded.

### Pretreatment

Before participation in the study, subjects received patient information and signed and dated the study consent form, which the Ethics Committee had approved. Before treatment, subjects received a brief general examination including medical history and survey of current medications. Pretreatment photographs of the NLFs were taken for each subject and used throughout the course of the study to assist the subject and investigator in completion of the Global Aesthetic Improvement Scale (GAIS) at the follow-up visits. The investigator assessed and recorded initial wrinkle severity of both NLFs using the WSRS.

### Treatment

Before the treatment, randomization of the PCL-1 or PCL-2 formula was determined and the applicable PCL formula administered to eligible subjects in both NLFs in accordance with the instructions for use. The PCL product formulas do not contain an anesthetic. The use of topical or local anesthesia was permitted at the discretion of the investigator. Approximately half of all subjects (55%) requested anesthesia. The PCL-1 or PCL-2 injection was administered into the deep dermis using a 27-G needle parallel to the length of the wrinkle or fold using a retrograde injection technique. Subjects were initially given a suboptimal dose, and the investigators were allowed to provide a touch-up at the 1-month follow-up visit. After administration, the injection site was gently massaged. Subjects were asked to return to the study site 1, 3, 6, 9, 12, 15, 18, and 24 months after the initial treatment. Safety and efficacy were assessed during these visits using GAIS and WSRS ratings. At each visit, subjects completed a visual analog scale (VAS) questionnaire to record their level of satisfaction and likelihood to return (probability of returning for repeat treatment with the same product after completion of the study).

## Results

### Effectiveness

The WSRS and GAIS assessments were used to determine effectiveness. Subject-evaluated GAIS and VAS assessments were used to support these findings.

### WSRS Ratings

A repeated analysis of variance (R-ANOVA) model on the WSRS using SAS Proc Mixed (SAS Institute, Inc., Cary, NC) was applied to the data to account for within-subject correlation. The data were summarized for the total NLFs rather than according to face side because there were no statistical differences found when testing WSRS between the left and right side of the face. Month 6, 9, 12, 15, 18, and 24 data were pooled to a similar R-ANOVA model to investigate the WSRS effect trending with time after 3 months. After month 3, linear and quadratic time effects in the PCL-2 R-ANOVA model were tested and found not to be statistically significant. In the PCL-2 data, there were no detectable statistically significant differences in subjects over time, showing sustained performance for PCL-2 for 24 months (linear *p* = .52; quadratic *p* > .99). Running the same model for PCL-1 using data after month 3 up to 12 months showed no statistically significant differences for PCL-1, showing sustained performance for PCL-1 for 12 months (linear *p* = .24; quadratic *p* = .16).

WSRS improvement >1 through 12 and 24 months indicated sustained performance over time in PLC-1 and PCL-2, respectively, in at least 50% of the population, with no statistically significant difference over time for PCL-2 (linear *p* = .21; quadratic *p* = .19) and PCL-1 (linear *p* = .12; quadratic *p* = .12).

### GAIS Ratings

On the investigator-evaluated GAIS assessments, for PCL-2, 100% of subjects reported improvement at 24 months. For PCL-1, 90% of subjects reported improvement at 12 months, after which the GAIS evaluation for the PCL-1 arm decreased to 78% at 24 months. On GAIS, a R-ANOVA model using SAS Proc Mixed was applied to the data to account for within-subject correlation. Month 6, 9, 12, 15, 18, and 24 data were pooled to a similar R-ANOVA model to investigate the GAIS effect trending with time after 3 months. After month 3, linear and quadratic time effects in this R-ANOVA model were tested and found to be statistically significant in the PCL-2 group (linear *p* = .04; quadratic *p* = .02), but results in Table 1 suggest that, on average, total improvement for PCL-2 was maintained up to 24 months after injection.

**TABLE 1:** Investigator-Evaluated Global Aesthetic Improvement Scale (GAIS)

Product	GAIS Score Change	3 Months	6 Months	9 Months	12 Months	15 Months	18 Months	24 Months
PCL-1	*n*	18	20	20	20	20	18	18
	Very much improved, %	0.0	20.0	10.0	20.0	0.0	11.1	0.0
	Much improved, %	55.0	45.0	45.0	60.0	10.0	11.1	0.0
	Improved, %	35.0	15.0	35.0	10.0	40.0	44.4	77.8
	No change, %	10.0	20.0	10.0	10.0	50.0	33.3	22.2
	worse, %	0.0	0.0	0.0	0.0	0.0	0.0	0.0
	Total improved, %	90.0	80.0	90.0	90.0	50.0	50.0	77.8
PCL-2	*n*	58	58	58	58	54	54	56
	Very much improved, %	3.5	13.8	5.2	6.9	3.7	5.6	0.0
	Much improved, %	51.7	50.0	63.8	70.7	51.9	50.0	23.2
	Improved, %	41.4	25.9	27.6	13.8	37.0	44.4	76.8
	No change, %	3.5	10.3	3.5	8.6	7.4	0.0	0.0
	Worse, %	0.0	0.0	0.0	0.0	0.0	0.0	0.0
	Total improved, %	96.6	89.7	96.6	91.4	92.6	100.0	100.0

**Figure 1:**
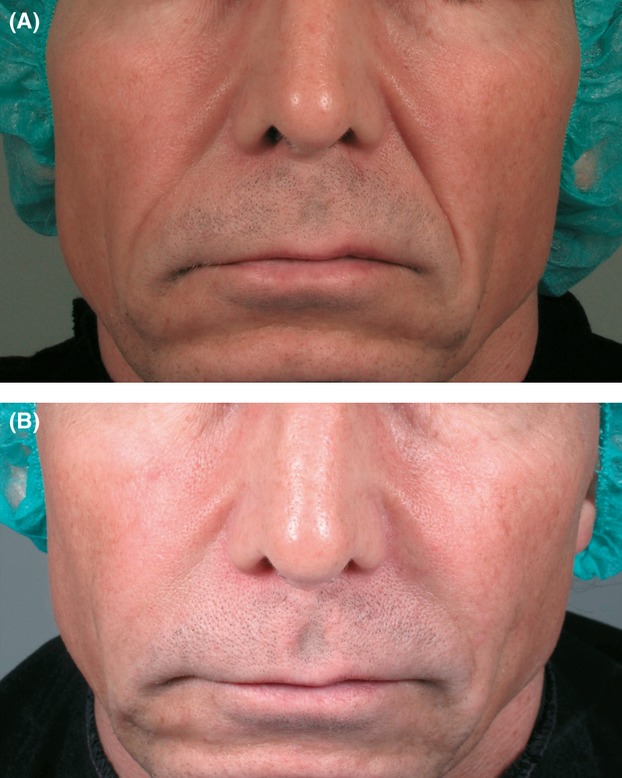
A 49-year-old man who received 1.5 mL of PCL-1 in the left and 1.0 mL of PCL-1 in the right nasolabial fold: (A) Baseline and (B) 12 months after initial injection. Global Aesthetic Improvement Scale ratings at 12 months were much improved.

**Figure 2:**
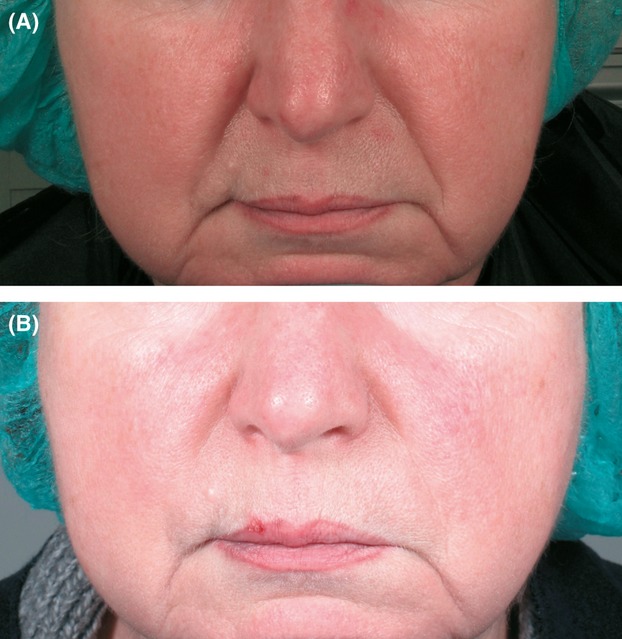
A 57-year-old woman who received 1.3 mL of PCL-2 in the left and 1.4 mL of PCL-2 in the right nasolabial fold: (A) Baseline and (B) 24 months after initial injection. Global Aesthetic Improvement Scale ratings at 24 months were much improved.

After 3 months, linear and quadratic time effects in the PCL-1 R-ANOVA model were tested using data from 6 to 12 months and were found not to be statistically significantly different for PCL-1 (linear *p* = .24; quadratic *p* = .20). Subject evaluations of GAIS ratings for PCL-1 (linear *p* = .41; quadratic *p* = .29) and PCL-2 (linear *p* = .68; quadratic *p* = .94) were also found to support sustained performance findings. Representative photographs of the treated NLFs are shown in Figures 1 and 2.

### Patient Satisfaction

Patient satisfaction was recorded using a VAS questionnaire. Patients were asked about their overall satisfaction with treatment results and the likelihood of returning for regular treatment with the product injected into their NLFs.

Subjects rated their satisfaction at 24 months as 81.7% for PCL-2 and 72.4% for PCL-1. At 24 months, subjects treated with PCL-2 were 78.0% likely to return for additional treatments on average, and subjects treated with PCL-1 were 75.3% likely to return at 24 months (Figures 1 and 2).

### Injected Volumes

Volumes at the initial suboptimal injection and at the 1-month touch-up are shown in Table 2. All nine patients in the PCL-1 group and 27 of 30 patients in the PCL-2 group (90.0%) received a touch-up, as expected because of the initial conservative treatment.

**TABLE 2:** Total Average Volumes per Patient (Including Touch-Up)

	Initial Treatment	Touch-Up Treatment	Total Average Volume
	
Product	mL		
Polycaprolactone-1	1.36	0.70	2.06
Polycaprolactone-2	1.20	0.76	1.96

No injections were offered after the 1-month touch-up. Total mean injected volumes used for the initial and touch-up injections were 1.96 mL for PCL-2 and 2.06 mL for PCL-1 (Table 2).

### Safety

No serious adverse events were reported at any of time points. Reported injection-related adverse events, such as edema (12 mild [30%] and 1 moderate [4%]) and ecchymosis (2 mild [5%]), all resolved without intervention. No nodules, granulomas, or other complications were reported. PCL-1 and PCL-2 are both considered to be safe and well tolerated.

## Discussion

### Satisfaction

Subject ratings demonstrated high and consistent satisfaction for both product formulations throughout the duration of the 24-month study. Investigation of likelihood to return for an additional treatment at 24 months also showed consistently high ratings for both formulations, suggesting high satisfaction and readiness for repeat treatments for both product formulations.

### Duration

Sustained performance (consistent and continued improvement over time) was demonstrated using the WSRS results. Longer-lasting sustained performance was found for PCL-2 than PCL-1. Investigator GAIS results confirmed these results, with patient GAIS results supporting the investigator findings. At each point during the study, consistent and continued within-subject performance was statistically demonstrated for PCL-1 to 12 months and for PCL-2 to 24 months.

GAIS assessments found 100% of subjects showing improvement in wrinkle severity at 24 months for PCL-2 and 90% of subjects showing improvement at 12 months for PCL-1. Subject and investigator GAIS assessments indicate sustained within-subject improvement and performance for PCL-1 12 months and PCL-2 for 24 months.

### Volume

Average total volume used for two NLFs for both formulations for initial treatment and maintenance of improvement was 2.06 mL for PCL-2 and 1.96 mL for PCL-1, confirming a consistent formulation of both products and an effective filling capacity for immediate and sustained improvement for their respective durations.

### Cost

Cost-minded physicians and subjects may appreciate the demonstrated feature of sustained performance providing continued and cost-effective improvement over time, with regard to currently available dermal fillers.

## Conclusions

PCL-1 and PCL-2 had comparable safety profiles and are safe and well tolerated. PCL-2 outperforms PCL-1 in duration of sustained performance as a result of the developed STAT technology.

Different PCL formulations provide a choice to physicians and subjects for desired and optimal duration of effect and associated sustained performance for physician, subject, and application requirements.

We hypothesize that, because of an induced neocollagenesis process, in combination with the specific bioresorption time of the selected PCL-formulation, this PCL-based filler leads to longer-lasting results than current, conventional dermal fillers provide.

We believe that this new dermal filler family will provide a unique option for patients seeking longer-lasting but nonpermanent results.
